# A Sensory-Centered Logistic–Arrhenius Framework for Shelf-Life Prediction of *Flammulina filiformis* Under Different Storage Temperatures

**DOI:** 10.3390/foods15132276

**Published:** 2026-06-25

**Authors:** Yongsheng Ma, Zhiyu Han, Ying Zhang, Shuai Xu, Changtian Li, Yu Li

**Affiliations:** Engineering Research Center of Edible and Medicinal Fungi, Ministry of Education, Jilin Agricultural University, Changchun 130118, China; mys@mails.jlau.edu.cn (Y.M.); zhiyuhanjlau@126.com (Z.H.); zhang1998a@163.com (Y.Z.); yuli966@126.com (Y.L.)

**Keywords:** shelf-life prediction, sensory evaluation, logistic regression model, arrhenius temperature modeling

## Abstract

Fresh edible mushrooms deteriorate rapidly during distribution, leading to quality loss, retail rejection, and avoidable waste of product, packaging, and refrigeration resources. Here, we developed a probability-based sensory shelf-life framework for commercially packaged Flammulina filiformis under controlled storage. Three hundred retail packages were stored at 4, 15 and 25 °C. Four sensory defects were scored and integrated into a composite overall quality index (OQ), and the endpoint attainment probability, *p* (OQ ≥ 3), was modeled by temperature-specific logistic regression. Whiteness, weight loss, polyphenol oxidase (PPO) activity, malondialdehyde (MDA) content, and soluble protein content were measured as supporting quality indices. Sensory rejection increased progressively and was accelerated at higher temperatures. Off-odor emerged earlier than the other defects and governed overall acceptability. Logistic models closely tracked endpoint progression and estimated shelf lives of 31.9 h (25 °C), 104.7 h (15 °C), and 261.4 h (4 °C), with relative errors within 3% compared with observed values. This sensory-centered framework provides an interpretable basis for shelf-life prediction and quality management of packaged enoki mushrooms.

## 1. Introduction

Edible macrofungi are widely consumed and are sold mainly as fresh, often packaged, products or as dried products [1]. Drying confers long storage stability and reduces postharvest losses by slowing biochemical and microbial deterioration [2]. Fresh mushrooms, by contrast, have high moisture content and remain metabolically active after harvest, which makes them highly perishable during storage and distribution [3]. Their short shelf life causes quality loss, product rejection, and avoidable waste of biological material, packaging, and cold-chain inputs. More reliable shelf-life evaluation for fresh packaged mushrooms is therefore important for both resource efficiency and market management.

**Figure 1:**
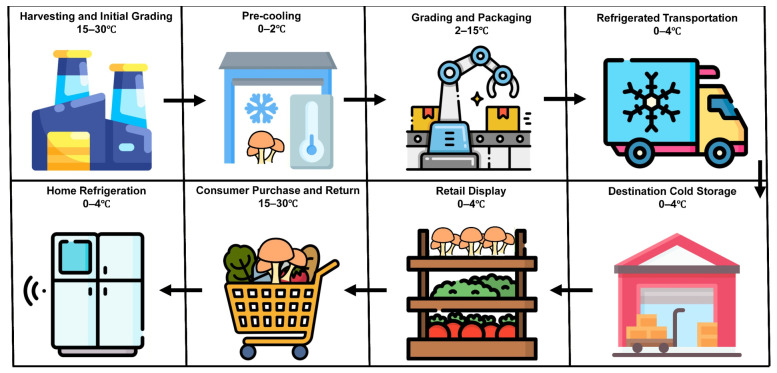
Cold-chain distribution pathway and key temperature nodes for domestically produced edible macrofungi. Arrows indicate each step of the mushroom cold-chain circulation.

Temperature is a major factor controlling postharvest deterioration of fresh edible mushrooms [4]. In commercial circulation, packaged mushrooms are exposed to multiple thermal environments rather than a single constant temperature [5]. As shown in Figure 1, products typically pass through harvesting and initial grading, pre-cooling, grading and packaging, refrigerated transportation, destination cold storage, retail display, consumer purchase/return transport, and home refrigeration. Although refrigeration is used in several stages, short-term warm exposure commonly occurs during handling and consumer transport, which can accelerate moisture loss, browning-related changes, texture deterioration, and odor defects, thereby shortening shelf life and increasing quality variability.

A broad range of strategies has been used to extend mushroom shelf life, including low-temperature storage, pre-cooling, modified-atmosphere or moisture-control packaging, washing and sanitizing treatments, anti-browning interventions, and antioxidant treatments [6,7]. These approaches can delay deterioration by slowing discoloration, reducing water loss, suppressing microbial growth, or maintaining texture-related quality [3]. However, their effects are often assessed using individual physicochemical or microbiological indicators under controlled storage conditions, and the chosen shelf-life endpoint is not always directly linked to practical rejection of packaged products.

This limitation is important for commercially marketed packaged mushrooms, for which rejection is driven mainly by sensory defects and may occur when a single severe defect becomes apparent [8]. Sensory evaluation is therefore more directly related to marketability than any single quality index. Common threshold-based approaches, such as the first time point at which a mean sensory score crosses a cutoff, reduce sample and panel variability to one average value and treat rejection as a discrete event. In practice, rejection develops progressively, and appearance-, texture- and odor-related defects do not necessarily progress at the same rate. Kinetic shelf-life models based on quality-index degradation or specific spoilage organism growth are useful for process description [9], but their endpoints may not match consumer-facing rejection when acceptability depends on combined sensory defects. A probability-based sensory framework can address this issue by linking prediction directly to sensory rejection, representing rejection as a progressive process and integrating multiple defect dimensions. Logistic modeling is well-suited to time-dependent endpoint attainment probability [10], and Arrhenius analysis can quantify the temperature dependence of the apparent deterioration rate parameter [11].

Accordingly, this study used commercially marketed packaged *Flammulina filiformis* (enoki mushroom) as a representative fresh mushroom product to develop a probability-based sensory shelf-life prediction framework. Four sensory attributes, namely cap color yellowing/dulling, cap integrity, surface sliminess/exudate, and off-odor, were evaluated and integrated into composite sensory indices representing appearance/tactile deterioration and overall acceptability. Sensory rejection endpoints were converted into endpoint attainment probabilities and fitted with temperature-specific logistic models. The resulting logistic rate constants were then related to temperature using an Arrhenius relationship to establish a Logistic–Arrhenius shelf-life prediction model.

## 2. Materials and Methods

### 2.1. Experimental Materials and Data Collection

A total of 300 retail packages of enoki mushroom (*Flammulina filiformis*) were obtained from Changchun Gaorong Biotechnology Co., Ltd, Changchun, China. All packages met the manufacturer’s commercial quality-control standards and were eligible for supermarket sale. The products were transported directly from the factory to the laboratory by refrigerated truck under cold-chain conditions. All samples came from a single commercial production and packaging batch (2 January 2026). Packages remained unopened in their original retail packaging throughout the experiment to preserve the market-sale condition; the packaging type was recorded as the manufacturer’s standard retail package for enoki mushrooms.

On arrival, samples were randomly assigned to three storage-temperature groups: 4, 15, and 25 °C. Relative humidity was controlled at 85–90% RH to simulate routine retail conditions. Sampling followed the predefined schedule for each temperature condition: every 48 h at 4 °C, every 24 h at 15 °C, and every 12 h at 25 °C. The detailed sampling framework is provided in Table S1.

### 2.2. Sensory Evaluation

Sensory evaluation was conducted under simulated supermarket display conditions using packaged *F. filiformis* stored at 4, 15, and 25 °C, with sampling intervals of 48, 24, and 12 h, respectively. At each temperature-by-time point, three independent package replicates were prepared.

The sensory panel consisted of 15 semi-trained internal assessors from the Process Research Center of Edible and Medicinal Fungi, Jilin Agricultural University. Assessors were selected to ensure consistent participation across repeated evaluations throughout the full storage experiment and were familiar with sensory quality changes in edible mushrooms. Because the aim of this study was controlled shelf-life model calibration rather than consumer preference testing, a stable internal panel was used to reduce between-assessor variability across time points. The study was approved by the Ethics Committee of Jilin Agricultural University. Approval Code is SE20260102. All participants provided written informed consent; participation was voluntary, withdrawal was permitted at any time, and all records were anonymized.

Before formal testing, panelists received standardized training on the anchored 0–4 scales (Table S2) and completed pilot sessions to align scoring criteria. To improve consistency in odor assessment and reduce aroma-loss bias after package opening, off-odor was always scored first, immediately after opening, within a fixed short time window, and using a standardized sniffing procedure (fixed distance and sniff duration, as defined in the panel standard operating procedure). Independent packages were opened in parallel at each time point, and assessors were distributed across package replicates so that odor was evaluated from freshly opened samples. Visual and tactile attributes were scored after odor assessment.

Four sensory attributes were scored on an anchored 0–4 ordinal scale: cap color yellowing/dulling (A), cap integrity (B), surface sliminess/exudate (C), and off-odor (O). Detailed scoring criteria and representative visual references used for panel training and scoring alignment are provided in Table S2 and Figure S1.

### 2.3. Composite Sensory Indices (OVQ and OQ) and Shelf-Life Endpoint Definition

**Figure 2:**
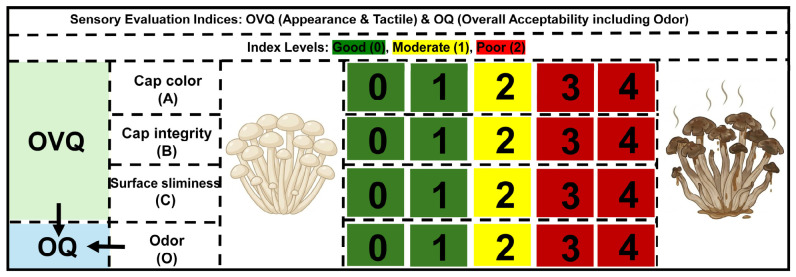
Schematic framework for sensory index construction and shelf-life endpoint definition. Four sensory attributes scored on a 0–4 scale—cap color yellowing/dulling (**A**), cap integrity (**B**), surface sliminess/exudate (**C**), and off-odor (**O**)—were integrated into two composite indices. ${\mathrm{OVQ}}^{\mathrm{raw}}$ was defined as max(*A*,*B*,*C*) (appearance/tactile quality), ${\mathrm{OQ}}^{\mathrm{raw}}$ was defined as max⁡(*A*,*B*,*C*,*O*) (overall sensory quality including odor). The shelf-life endpoint used for this study was defined as p(OQ≥3)≥0.5.

To distinguish appearance/tactile deterioration from overall sensory acceptability, two composite indices were defined from the four sensory attributes (A, B, C, and O; each scored 0–4) (Figure 2). OVQ, the appearance/tactile quality index, included the non-odor attributes of cap color yellowing/dulling (A), cap integrity (B), and surface sliminess/exudate (C). OQ, the overall quality index, included all four attributes and therefore represented consumer-facing quality, including odor. A worst-attribute rule was used in Equations (1)–(2).

(1) $$\begin{array}{c} OVQ_{i,r,T,t}^{raw}=max\left(A_{i,r,T,t},\;B_{i,r,T,t},\;C_{i,r,T,t}\right) \end{array}$$

(2) $$\begin{array}{c} OQ_{i,r,T,t}^{raw}=max\left(OVQ_{i,r,T,t}^{raw},\;O_{i,r,T,t}\right)=max\left(A_{i,r,T,t},\;B_{i,r,T,t},\;C_{i,r,T,t},\;O_{i,r,T,t}\right) \end{array}$$

where *i* denotes the panelist, *r* the package replicate, *T* the storage temperature, and *t* the storage time.

The maximum-value rule was chosen because, in retail practice, a single severe defect, such as a strong off-odor or severe sliminess, can make a package unacceptable even when other attributes remain acceptable. Thus, OVQ captures the worst appearance/tactile defect, whereas OQ captures the worst defect across all sensory dimensions.

For shelf-life modeling, a single endpoint was predefined as shown in Equation (3).

(3) $$\begin{array}{c} p(OQ\geq 3)\geq 0.5 \end{array}$$

That is, at least 50% of valid panelist scores at a given temperature-by-time point reached OQ ≥ 3 (equivalent to at least 8 of 15 scores when all 15 panelists were valid).

To avoid ambiguity, the 0–4 raw sensory scores and the color display in Figure 2 served different purposes. Raw scores of 0 and 1 indicate acceptable quality, 2 indicates moderate deterioration, and 3 and 4 indicate unacceptable deterioration. The colors in Figure 2 were used only to visualize these score ranges and were not treated as additional variables in the statistical model.

### 2.4. Whiteness

Whiteness was measured using an automatic portable whiteness meter (LC-WSB) and expressed as a percentage (%). At each temperature-by-time point, three independent freshly sampled packages were measured.

### 2.5. Weight Loss Rate

At day 0, all packages were coded, and their initial weights were recorded. At each temperature-by-time point, three packages were randomly selected and weighed once. Weight loss rate was calculated as shown in Equation (4) [12].

(4) $$\begin{array}{c} \mathrm{Weight}\;\mathrm{loss}\;\mathrm{rate}\;(\%)=\frac{W_{0}-W_{t}}{W_{0}}\times 100 \end{array}$$

where W_0_ is the initial package weight, and W_t_ is the package weight at sampling time t.

### 2.6. PPO Activity

Polyphenol oxidase (PPO) activity was determined using a commercial assay kit (Solarbio, Beijing, China) according to the manufacturer’s instructions. Absorbance was measured at 410 nm. One unit (U) was defined as a 0.01 change in absorbance per minute per gram of tissue in a 1 mL reaction system [13]. PPO activity was calculated as shown in Equation (5).

(5) $$\begin{array}{c} {\mathrm{PPO}\;\mathrm{activity}\;(U\;g}^{-1}\;\mathrm{fresh}\;\mathrm{weight})=\frac{60\times \Delta A}{W}\times F \end{array}$$

where ΔA denotes the change in absorbance per minute, W the sample mass (g), and F the dilution factor.

### 2.7. MDA Content

Malondialdehyde (MDA) content was measured using a commercial assay kit (Solarbio, Beijing, China), following the manufacturer’s instructions [14]. MDA was calculated on a sample-mass basis, as shown in Equation (6).

(6) $$\begin{array}{c} {\mathrm{MDA}\;\mathrm{content}\;(\mathrm{nmol}\;g}^{-1}\;\mathrm{fresh}\;\mathrm{weight})=32.258\times \frac{\Delta A}{W}\times F \end{array}$$

where ΔA = ΔA_532_ − ΔA_600_, *W* is sample mass (g), and *F* is dilution factor.

### 2.8. Soluble Protein Content

Soluble protein was determined by the Coomassie Brilliant Blue (Bradford) method. Bovine serum albumin (BSA) was used for calibration $\left(y=0.0499x+0.0192\right)$. Approximately 2.0 g of *F. filiformis* was homogenized with 5 mL of distilled water for 3 min, centrifuged at 12,000× *g* for 20 min at 4 °C, and the supernatant was mixed with 5 mL of Coomassie reagent. Absorbance was measured at 595 nm [15]. Soluble protein content was calculated as shown in Equation (7).

(7) $$\begin{array}{c} {\mathrm{Soluble}\;\mathrm{protein}\;(g\;\mathrm{kg}}^{-1}\;\mathrm{fresh}\;\mathrm{weight})=\frac{\left(A_{595}-0.0192\right)}{0.0499}\times \frac{V_{\mathrm{extract}}\times F}{W} \end{array}$$

where *A*_595_ is absorbance at 595 nm, *V_extract_* is extraction volume (mL), *F* is dilution factor, and *W* is sample mass (g).

### 2.9. Logistic Kinetic Modeling and Shelf-Life Estimation

To model shelf-life progression, the proportion of panelist scores meeting the predefined endpoint *p* (OQ ≥ 3) at each sampling time was used as the response variable. Storage time (t, h) was used as the predictor, and a separate logistic regression model was fitted for each storage temperature using a binomial distribution with a logit link function, as shown in Equation (8).

(8) $$\begin{array}{c} ln\left(\frac{p}{1-p}\right)=\beta_{0}+kt \end{array}$$

where *p* = *p* (*OQ* ≥ *3*) is the deterioration probability (i.e., the probability that the composite sensory score reached or exceeded 3), *β*_0_ is the intercept, and k(h^−1^) is the apparent rate constant.

When *p* = 0.5, the shelf-life endpoint is reached (i.e., at least 50% of valid panelist scores satisfy *OQ* ≥ 3). Substituting *p* = 0.5 into Equation (9) gives the theoretical shelf life (SL):

(9) $$\begin{array}{c} SL=-\frac{\beta_{0}}{k} \end{array}$$

The models were fitted in R using the glm() function with a binomial family and logit link. Model fit quality at each temperature was evaluated using the Akaike information criterion (AIC). The 95% confidence intervals of predicted values were obtained from the predict() function with se.fit = TRUE and calculated as fitted value ±1.96 × SE.

### 2.10. Arrhenius Relationship and Apparent Activation Energy Estimation

The temperature dependence of the apparent rate constant (k) obtained from the logistic models was described using the Arrhenius equation as shown in Equation (10).

(10) $$\begin{array}{c} k=k_{0}\cdot exp\left(-\frac{Eₐ}{RT}\right) \end{array}$$

where k is the apparent rate constant (h^−1^), k0 is the pre-exponential factor, Ea is the apparent activation energy (kJ mol^−1^), T is absolute temperature (K), and R is the universal gas constant (8.314 J mol^−1^ K^−1^).

By taking the natural logarithm, Equation (11) was linearized as:

(11) $$\begin{array}{c} ln(k)=ln(k_{0})-\frac{E_{a}}{R}\left(\frac{1}{T}\right) \end{array}$$

A linear regression was performed using 1/*T* as the independent variable and ln (k) as the dependent variable. The apparent activation energy (E_a_) was estimated from the slope ($-E_{a}/R$), and the corresponding 95% confidence interval was reported.

Combining the Arrhenius relationship with the logistic model intercept (*β*_0_), the shelf life at a given temperature *T* was predicted as shown in Equation (12).

(12) $$\begin{array}{c} SL(T)=-\beta_{0}/[k^{0}\cdot exp(-\frac{Eₐ}{RT})] \end{array}$$

where *SL*(*T*) is the predicted shelf life (h).

### 2.11. Model Validation and Uncertainty Analysis

The generalization performance of the Arrhenius-based shelf-life prediction model was evaluated using leave-one-out cross-validation (LOO-CV). Because only three calibration temperatures were available, one temperature point was removed in each iteration, the Arrhenius relationship was refitted using the remaining two temperature points, and shelf life at the omitted temperature was predicted. Prediction error was then calculated by comparing the predicted shelf life ($SL_{pred}$) with the observed shelf life ($SL_{obs}$) at the omitted temperature.

Model performance was assessed using the mean absolute error (MAE), root mean square error (RMSE), bias factor ($B_{f}$), and accuracy factor ($A_{f}$), as shown in Equations (13)–(16).

(13) $$\begin{array}{c} MAE=\frac{1}{n}\sum_{i=1}^{n}|SL_{pred,i}-SL_{obs,i}| \end{array}$$

(14) $$\begin{array}{c} RMSE=\sqrt{\frac{1}{n}\sum_{i=1}^{n}{\left(SL_{pred,i}-SL_{obs,i}\right)}^{2}} \end{array}$$

(15) $$\begin{array}{c} B_{f}=10^{\frac{1}{n}\sum_{i=1}^{n}{log}_{10}\left(\frac{SL_{pred,i}}{SL_{obs,i}}\right)} \end{array}$$

(16) $$\begin{array}{c} A_{f}=10^{\frac{1}{n}\sum_{i=1}^{n}|{log}_{10}\left(\frac{SL_{pred,i}}{SL_{obs,i}}\right)|} \end{array}$$

where *n* is the number of validation predictions. A $B_{f}$ value greater than 1 indicates systematic overestimation of shelf life (less conservative prediction), whereas $B_{f}<1$ indicates underestimation (more conservative prediction). The $A_{f}$ value reflects the average deviation between predicted and observed values, with values closer to 1 indicating higher predictive accuracy.

Bootstrap resampling (1000 iterations) was also used to quantify parameter uncertainty. Percentile-based 95% confidence intervals were estimated for the apparent activation energy (Ea) and predicted shelf-life values at each temperature.

### 2.12. Statistical Analysis

Objective quality indices (whiteness, weight loss, PPO activity, MDA content, and soluble protein content) were analyzed within each storage temperature using one-way ANOVA with storage time as the factor. Normality of residuals and homogeneity of variance were checked using the Shapiro–Wilk and Levene’s tests, respectively; when parametric assumptions were not met, the Kruskal–Wallis test was used as a non-parametric reference. Spearman’s rank correlation was used to assess associations between mean OQ and objective indices, and multiple linear regression was performed with mean OQ as the dependent variable and whiteness, weight loss, PPO activity, MDA content, and soluble protein content as predictors. All data analysis and figure generation were performed in R, and statistical significance was set at *p* < 0.05.

## 3. Results and Discussion

### 3.1. Temperature-Dependent Deterioration Fingerprints and Overall Sensory Quality Evolution

**Figure 3:**
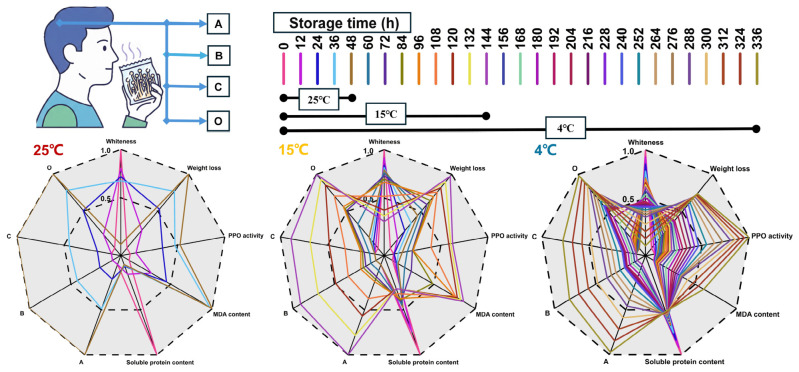
Integrated deterioration fingerprints of *F. filiformis* under different storage temperatures. The figure shows the sensory evaluation framework (**A**, **B**, **C**, and **O**), storage timelines (4, 15, and 25 °C), and spider plots combining sensory attributes with physicochemical indicators (whiteness, weight loss, PPO activity, MDA content, and soluble protein content). Colored trajectories represent storage time points, and the top color scale indicates storage time (h). Values displayed in the spider plots were linearly interpolated between measured time points for visualization only (see Supplementary Methods). All variables were min–max normalized (0–1).

To enable temporal comparison across storage temperatures with different sampling intervals, continuous daily values shown in the spider plot were generated by linear interpolation between measured time points, while preserving the observed trends [12]. All indicators were scaled to 0–1 using min–max normalization for visualization (see Supplementary Methods). Figure 3 provides an integrated overview of temperature-dependent quality deterioration in *Flammulina filiformis* using a normalized fingerprint (spider) plot that combines sensory attributes (A, B, C, and O) and physicochemical indicators.

Clear temperature-dependent differences were observed in both the rate and trajectory of quality change. The 25 °C group showed the most rapid shift in the fingerprint pattern over a short storage period, indicating accelerated overall deterioration. In contrast, samples stored at 4 °C exhibited a much slower and more gradual evolution over an extended storage period, while the 15 °C group showed an intermediate pattern. Importantly, the fingerprint plot shows that deterioration was a multi-attribute process rather than a change in a single variable.

### 3.2. Time-Course Changes in Sensory Attributes, OVQ/OQ, and Objective Quality Indicators

**Figure 4:**
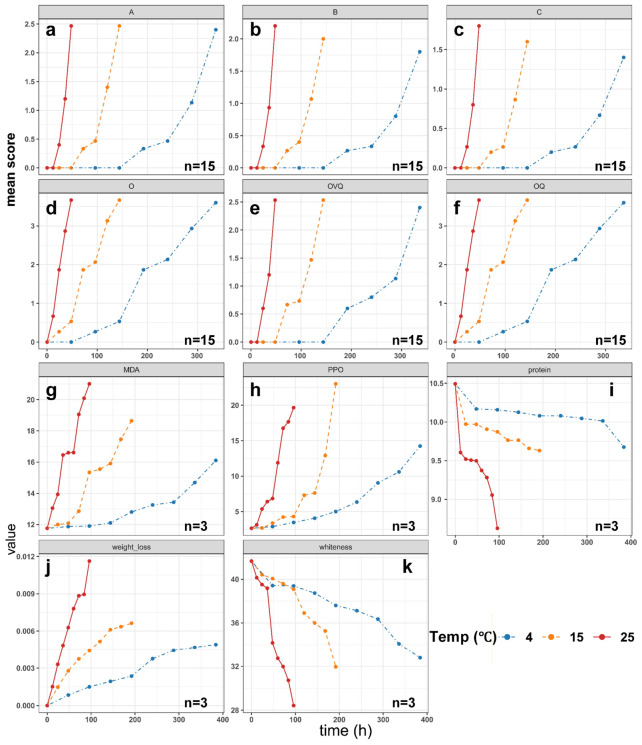
Time-course changes in sensory attributes, composite sensory indices, and objective quality indicators of *F. filiformis* during storage at different temperatures. Changes in sensory attribute scores and objective quality indicators during storage at 4, 15, and 25 °C. Panels show: (**a**) A (cap yellowing/dulling), (**b**) B (cap integrity), (**c**) C (surface sliminess/exudation), (**d**) O (off-odor), (**e**) OVQ (appearance/texture-related composite score), (**f**) OQ (overall quality score, defined by the max-rule), (**g**) MDA content, (**h**) PPO activity, (**i**) soluble protein content, (**j**) weight loss, and (**k**) whiteness. Blue, orange, and red lines represent storage at 4, 15, and 25 °C, respectively. Lines connect observed time points to visualize temporal trends.

Figure 4 summarizes the time-course evolution of sensory attributes (A, B, C, and O), composite sensory indices (OVQ and OQ), and objective quality indicators of *F. filiformis* during storage at 4, 15, and 25 °C. Sensory scoring was performed up to 48 h (25 °C), 144 h (15 °C), and 336 h (4 °C), whereas objective indicators (whiteness, weight loss, PPO activity, MDA content, and soluble protein content) were measured at nine sampling points within each temperature-specific storage period (25 °C: 0–96 h; 15 °C: 0–192 h; 4 °C: 0–384 h).

As shown in Figure 4a–f, all sensory defect scores increased with storage time, indicating progressive deterioration in appearance, surface condition, and odor. The temperature effect was clear: sensory changes developed earlier and more rapidly at higher temperatures. Among the four sensory attributes, off-odor (O) increased earlier and to a greater extent than the others. Under the max-rule definition, OQ equaled the maximum among A, B, C, and O at each time point, and OQ matched the off-odor score throughout storage, indicating that off-odor was the dominant factor controlling overall acceptability under the tested conditions.

The mean OQ reached ≥3 at 48 h (25 °C), 120 h (15 °C), and 336 h (4 °C), showing a clear temperature-dependent delay in sensory unacceptability. OVQ (appearance/texture-related composite) increased more slowly than OQ and remained lower than OQ at the last sensory time point in each temperature group, further supporting the view that odor deterioration was the limiting factor in overall sensory acceptance in this study.

Under the present storage conditions, OQ closely overlapped with the off-odor score, indicating that odor was the earliest limiting attribute in this dataset. Even so, the composite index retains practical value because it provides a unified framework-level endpoint that remains applicable when the dominant defect changes across batches, package systems, or storage environments. In the present product and packaging system, off-odor was the main driver of rejection; under other conditions, appearance- or surface-related defects may become limiting earlier.

The objective indicators changed in directions consistent with sensory deterioration (Figure 4g–k). Whiteness decreased significantly with storage time at all temperatures (25 °C, *p* < 0.001; 15 °C, *p* = 0.001; 4 °C, *p* = 0.002), declining from 41.67 initially to 28.42 (25 °C, 96 h), 31.96 (15 °C, 192 h), and 32.79 (4 °C, 384 h). This decline is consistent with the visual yellowing/dulling captured by sensory attribute A. In mushrooms, loss of whiteness is commonly associated with surface discoloration and browning-related reactions [16], which can be accelerated by higher temperature through increased metabolic activity and faster enzyme-mediated oxidation.

Weight loss increased significantly at all temperatures (all *p* < 0.001), reaching 0.0116, 0.00663, and 0.00490 at the final time points for 25, 15, and 4 °C, respectively (approximately 1.16%, 0.66%, and 0.49%). Weight loss mainly reflects water loss during storage. Increased water loss can reduce tissue turgor [17], weaken surface freshness, and promote deterioration in texture and visual quality, thereby contributing to the increases in B and C scores (cap integrity loss and surface sliminess/exudation-related defects).

PPO activity increased significantly over time in all temperature groups (all *p* < 0.001). PPO is a key enzyme involved in enzymatic browning [18], and its increasing activity is in line with the observed decline in whiteness and deterioration in visual appearance.

MDA content also increased significantly at all temperatures (all *p* < 0.001), indicating progressive membrane lipid peroxidation during storage. As a marker of oxidative damage, increased MDA suggests a gradual loss of membrane integrity [19].

Soluble protein content showed an overall decreasing trend, but the time effect was significant only at 25 °C (*p* < 0.001), and not significant at 15 °C (*p* = 0.356) or 4 °C (*p* = 0.224). This suggests that soluble protein was relatively stable at lower temperatures within the tested storage periods, but was more affected under warm storage. The reduction in soluble protein at 25 °C may reflect accelerated protein degradation and/or increased utilization of soluble nitrogenous compounds during postharvest metabolism [20].

### 3.3. Associations Between OQ and Objective Quality Indicators

**Figure 5:**
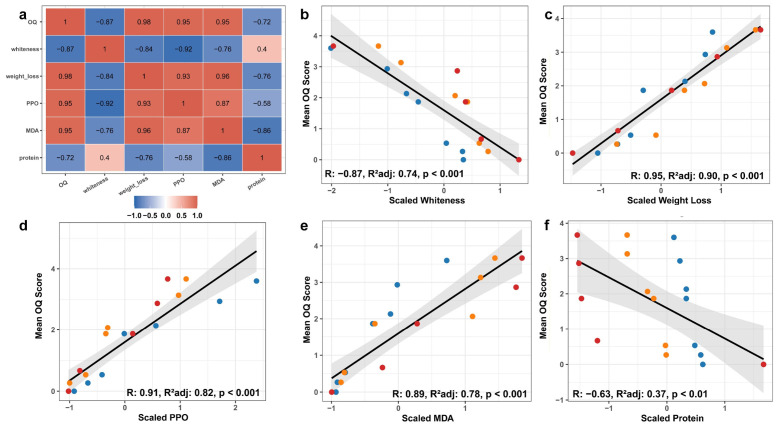
Correlation and regression analysis between mean OQ and objective quality indicators. (**a**) Spearman correlation heatmap showing pairwise correlations among mean OQ and the five objective indicators. (**b**–**f**) Linear regression plots of mean OQ versus scaled whiteness, weight loss, PPO activity, MDA content, and soluble protein content (pooled data from 4, 15, and 25 °C). Points are colored by storage temperature (blue, 4 °C; orange, 15 °C; red, 25 °C); black lines indicate fitted trends and shaded areas indicate 95% confidence intervals. Scatterplots use scaled variables for visualization only; the multivariable regression equation in the main text was fitted using original values.

As shown in the Spearman correlation heatmap (Figure 5a), mean OQ was highly positively correlated with weight loss (ρ = 0.976, *p* < 0.001), PPO activity (ρ = 0.951, *p* < 0.001), and MDA content (ρ = 0.946, *p* < 0.001), and highly negatively correlated with whiteness (ρ = −0.868, *p* < 0.001). Mean OQ was also negatively correlated with soluble protein content (ρ = −0.716, *p* < 0.001), although the association was weaker than those observed for weight loss, PPO, MDA, and whiteness. These results indicate that higher sensory deterioration (higher OQ) was consistently associated with greater moisture loss, stronger browning/oxidative responses, and lower visual whiteness.

The pairwise relationships shown in Figure 5b–f further support these trends. Mean OQ decreased with increasing whiteness, and increased with weight loss, PPO activity, and MDA content, with relatively strong linear trends across the pooled data points. By contrast, the association between mean OQ and soluble protein was weaker and more dispersed, which is consistent with the lower correlation coefficient observed in the heatmap. In addition, whiteness and soluble protein were only weakly correlated with each other (ρ = 0.396, *p* = 0.084), suggesting that these two indicators may reflect partly independent aspects of quality deterioration. Weight loss also showed strong positive correlations with PPO (ρ = 0.926) and MDA (ρ = 0.958), indicating coordinated progression of water loss and oxidative stress-related changes during storage.

To further evaluate the combined explanatory value of the objective indicators, a multiple linear regression model was fitted with mean OQ as the response variable and the five objective indicators as predictors: mean OQ = −4.427 − 0.020 × whiteness + 264.259 × weight loss + 0.230 × PPO + 0.227 × MDA + 0.180 × soluble protein.

The model showed excellent overall fit (R^2^ = 0.977, adjusted R^2^ = 0.968, F (5,14) = 117, *p* = 6.58 × 10^−11^), indicating that the five objective indicators jointly explained 97.7% of the variance in mean OQ. Among individual predictors, weight loss (*p* = 0.013), PPO activity (*p* = 0.001), and MDA content (*p* = 0.014) were significant contributors, whereas whiteness (*p* = 0.743) and soluble protein (*p* = 0.572) were not significant in the multivariable model.

### 3.4. Probability-Based Sensory Endpoint Modeling and Temperature Dependence of Shelf Life

#### 3.4.1. Logistic Modeling of Endpoint Attainment Probability and Shelf-Life Estimation

**Figure 6:**
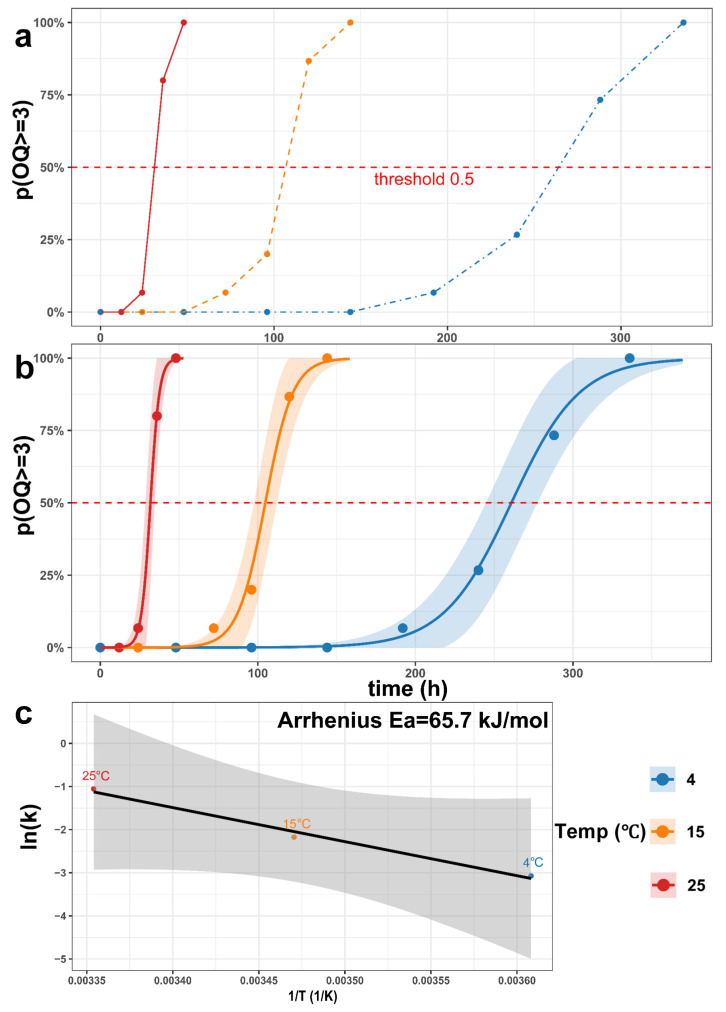
Probability-based sensory endpoint modeling and Arrhenius analysis for shelf-life prediction of *F. filiformis*. (**a**) Observed endpoint attainment probability, p(OQ≥3), as a function of storage time at 4, 15, and 25 °C. The dashed red horizontal line indicates the probability threshold of 0.5 used to define the sensory shelf-life endpoint. (**b**) Temperature-specific logistic fits of endpoint attainment probability over time, with shaded bands representing 95% confidence intervals. Points represent observed proportions at each sampling time. Shelf life (SL) was defined as the time corresponding to p(OQ≥3)=0.5. (**c**) Arrhenius plot of the logistic rate constant (ln*k*) versus reciprocal absolute temperature (1/T, K^−1^). The solid line indicates the linear regression fit, and the shaded band indicates the 95% confidence interval. The apparent activation energy ($E_{a}$) was estimated from the fitted slope. Blue, orange, and red represent storage at 4, 15, and 25 °C, respectively.

To quantify the progression of sensory failure over time, temperature-specific logistic models were fitted to the endpoint attainment probability, p(OQ≥3), using storage time as the predictor. The observed endpoint proportions and fitted curves (with 95% confidence bands) are shown in Figure 6a,b. At all three temperatures, $p\left(OQ\geq 3\right)$ increased with time in a sigmoidal manner, which is consistent with the expected transition from an acceptable state (low endpoint probability) to an unacceptable state (high endpoint probability) during storage [21].

The logistic curves closely tracked the observed endpoint proportions at 25, 15, and 4 °C (Figure 6b), indicating that the probability-based framework adequately described the time-dependent increase in sensory rejection under the tested conditions. This modeling strategy is useful because it converts panel-based sensory outcomes into a continuous probability function, rather than treating shelf life as a single discrete time point only. As a result, it better accommodates inter-evaluator variability and the gradual nature of sensory deterioration [22].

The estimated model parameters are summarized in Table 1. The fitted intercept term ($\beta_{0}$) varied only slightly across temperatures (−11.081 to −12.066), whereas the rate constant k showed a strong temperature dependence, increasing from 0.0462 h^−1^ at 4 °C to 0.1137 h^−1^ at 15 °C and 0.3478 h^−1^ at 25 °C. The k value at 25 °C was approximately 7.5 times that at 4 °C, showing that warm storage substantially accelerated the progression toward sensory unacceptability.

From the fitted logistic parameters, the theoretical shelf life was calculated as $SL=-\beta_{0}/k$, corresponding to the time at which p(OQ≥3)=0.5. The estimated values were 31.9 h at 25 °C, 104.7 h at 15 °C, and 261.4 h at 4 °C (Table 1). These model-derived values were very close to the observed shelf-life values (31.1, 106.9, and 264.0 h at 25, 15, and 4 °C, respectively), with absolute relative deviations of approximately 2.6%, 2.1%, and 1.0% (all within 3%). The close agreement supports the practical suitability of the endpoint probability definition and the logistic formulation for estimating shelf life in this product and packaging system [23]. This agreement is important. First, it indicates that the selected sensory endpoint criterion (OQ-based and probability-based) is internally consistent with the observed rejection process [22]. Second, it suggests that the fitted logistic curves are not only statistically reasonable but also practically interpretable for shelf-life decision-making. In other words, the model does not merely fit the observed proportions; it also returns shelf-life estimates that closely match the actual endpoint timing observed in the sensory panel.

The AIC values of the temperature-specific logistic models were 8.86 (25 °C), 12.82 (15 °C), and 13.33 (4 °C). These values indicate an acceptable fit for all three temperature-specific models [24]. However, because each logistic model was fitted to a separate dataset with different time spans and point distributions, the AIC values are reported mainly for within-model goodness-of-fit description and rough comparison, rather than for strict ranking of model quality across temperatures [25].

#### 3.4.2. Arrhenius Analysis of Temperature Dependence and Basis for Cross-Temperature Prediction

To describe the temperature dependence of the deterioration rate in the logistic model, the fitted rate constants (*k*) were linked to temperature using the Arrhenius relationship [12]. As shown in Figure 6c, linear regression of $\ln\left(k\right)$ against 1/*T* (K^−1^) produced a negative slope (−7897.2 K) and an intercept of 25.361, indicating that the sensory deterioration rate increased as temperature increased. Based on the fitted slope, the apparent activation energy was estimated as 65.66 kJ·mol^−1^, and the frequency factor was 1.03 × 10^11^ h^−1^. The Arrhenius fit showed high goodness-of-fit (R^2^ = 0.988; adjusted R^2^ = 0.976), suggesting that the rate constants derived from the logistic model followed an approximately Arrhenius-type temperature dependence within the tested range (4–25 °C). The estimated *E_a_* indicates substantial temperature sensitivity of the endpoint progression rate [26].

The magnitude of the estimated *E_a_* (65.66 kJ·mol^−1^) falls within a typical range reported for food quality deterioration processes (commonly on the order of tens to low hundreds of kJ·mol^−1^), and is also broadly consistent with temperature-sensitive postharvest deterioration phenomena involving enzymatic and oxidative pathways [27].

The uncertainty analysis highlights this limitation clearly. Since the Arrhenius regression used only three temperature points, the linear model had only one degree of freedom, and the F-test *p*-value was 0.070, which did not reach the conventional significance threshold (*p* < 0.05). Accordingly, the regression-based 95% confidence interval for *E_a_* was wide (−27.07 to 158.38 kJ·mol^−1^), indicating substantial uncertainty if inference relies only on classical small-sample regression statistics.

In contrast, bootstrap resampling produced a narrower percentile-based 95% confidence interval for Ea of 54.32–79.84 kJ·mol^−1^, supporting the physical plausibility of the estimated temperature sensitivity while still indicating the need for additional calibration temperatures.

Because the Arrhenius analysis was calibrated with only three storage temperatures, the estimated Ea should be interpreted as a preliminary product-specific parameter rather than a robust universal constant. With such sparse calibration, small uncertainty in the fitted k values can substantially affect the Arrhenius slope, especially toward the low-temperature end, where shelf life is longer. Therefore, the current Arrhenius result is most useful for proof-of-concept and within-range prediction, and additional calibration temperatures are needed to improve parameter stability.

### 3.5. In-Sample Agreement, Leave-One-Temperature-Out Cross-Validation, and Uncertainty Analysis

**Figure 7:**
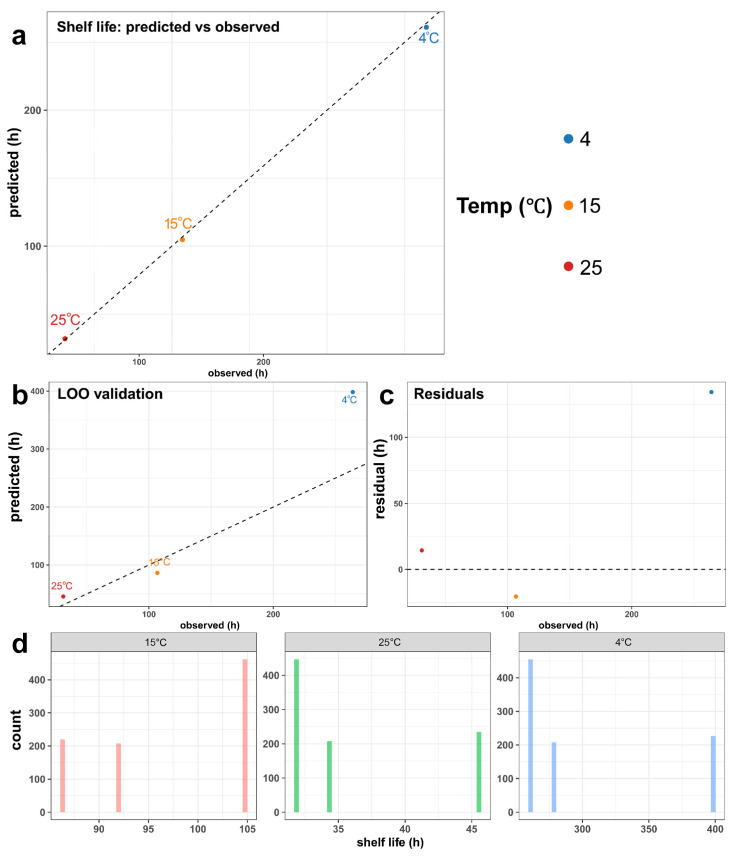
Predictive performance, leave-one-temperature-out cross-validation (LOO-CV), and bootstrap uncertainty of the Logistic–Arrhenius shelf-life model for *F. filiformis*. (**a**) Comparison between observed shelf life (SL) and model-predicted SL for the three storage temperatures (4, 15, and 25 °C); the dashed diagonal line indicates the 1:1 reference line. (**b**) Leave-one-temperature-out cross-validation (LOO-CV) results showing observed versus predicted SL when each temperature point was omitted in turn from Arrhenius calibration; the dashed diagonal line indicates the 1:1 reference line. (**c**) Residual plot of LOO-CV predictions (predicted SL−observed SL) as a function of observed SL; the dashed horizontal line indicates zero residual. (**d**) Bootstrap distributions of predicted SL (1000 resamples) for 4, 15, and 25 °C, showing the uncertainty of shelf-life estimates under each temperature condition. Points are colored by storage temperature (blue, 4 °C; orange, 15 °C; red, 25 °C). In panel (**d**), histograms are shown separately for each temperature.

The agreement between the observed shelf life, derived from measured data using linear interpolation, and the model-predicted shelf life is shown in Figure 7a and summarized in Table 2. Across the three calibration temperatures, the Logistic–Arrhenius model reproduced the observed shelf-life values with small deviations. The relative errors were −1.15% at 4 °C, −1.91% at 15 °C, and +2.49% at 25 °C, with a mean absolute error of approximately 1.95 h. These results indicate good internal consistency between the endpoint probability model and the Arrhenius-based temperature response. However, this comparison represents an in-sample agreement check rather than independent validation, because the same temperature points were used for model calibration.

Leave-one-temperature-out cross-validation (LOO-CV) was further used to evaluate cross-temperature prediction performance [28]. As shown in Figure 7b,c, the LOO-CV metrics were MAE = 56.41 h, RMSE = 78.87 h, bias factor (Bf) = 1.213, and accuracy factor (Af) = 1.398. The bias factor greater than 1 indicates an overall tendency to overpredict shelf life. The LOO-CV errors were larger than the in-sample errors, which is expected because only three constant-temperature points were available for Arrhenius calibration. In each LOO iteration, the Arrhenius relationship was refitted using only two temperatures, making the estimated slope highly sensitive to the retained data points. The largest absolute deviation occurred at 4 °C, where shelf life was much longer, and small kinetic uncertainty could result in large differences in predicted hours. Thus, the LOO-CV results should be viewed as a conservative indication of model stability under sparse temperature calibration.

Bootstrap resampling further characterized parameter-driven uncertainty in the shelf-life predictions [29]. As shown in Figure 7d, the bootstrap 95% confidence intervals were 261–398 h at 4 °C, 86–105 h at 15 °C, and 32–46 h at 25 °C. The absolute uncertainty increased as the temperature decreased, and the predicted shelf life became longer. This behavior is expected for Arrhenius-based shelf-life prediction, because uncertainty in kinetic parameters can be amplified when projected to lower temperatures, where the deterioration rate constant is smaller, and shelf life is longer [30]. Although the intervals showed different widths among temperatures, they remained physically plausible and preserved the expected shelf-life ranking of 4 °C > 15 °C > 25 °C. Therefore, prediction intervals are more appropriate than single-point estimates when the model is calibrated with a limited number of temperatures [31].

Overall, the fitted-data comparison, LOO-CV, and bootstrap analysis show that the Logistic–Arrhenius framework can describe temperature-dependent shelf-life changes in packaged *F. filiformis*, but its predictive certainty is limited by sparse temperature calibration. The current model should therefore be interpreted as a constant-temperature, product-specific shelf-life prediction framework. Further validation using more calibration temperatures, independent production batches, different packaging configurations, microbiological or volatile evidence, and dynamic cold-chain profiles is needed before broader industrial application.

From an application perspective, the present framework can support temperature-specific handling decisions for packaged enoki mushrooms under constant-temperature storage. Manufacturers may use the fitted shelf-life estimates to set internal holding limits and quality inspection schedules, distributors may use the temperature-dependent rate information to estimate remaining shelf life after warm exposure, and retailers may use the OQ-based endpoint as a practical trigger for stock rotation and display management. Although the current calibration is product- and packaging-specific, the same workflow can be recalibrated for other batches or packaging systems and may help support shelf-life labeling, inventory prioritization, and cold-chain quality control.

Microbiological measurements were not included in the present study, and therefore, the sensory endpoint should be interpreted as a marketability criterion rather than a microbiological safety limit. The increase in off-odor may be associated with microbial activity, but this mechanism was not directly verified here. Future work combining sensory scores with microbiological counts and/or volatile analysis would help clarify the biological basis of odor-driven rejection.

## 4. Conclusions

This study developed a probability-based sensory shelf-life prediction framework for *F. filiformis* by integrating a composite sensory endpoint (OQ-based rejection criterion) with Logistic and Arrhenius modeling. Storage temperature strongly affected deterioration dynamics: sensory defects, especially off-odor, increased much faster at higher temperatures, while whiteness decreased, and weight loss, PPO activity, and MDA content increased in parallel, indicating coordinated physical and biochemical deterioration. Correlation and regression analyses further showed that weight loss, PPO activity, and MDA content were the most sensitive objective indicators associated with OQ deterioration. Temperature-specific Logistic models successfully described the time-dependent increase in endpoint attainment probability, p(OQ≥3), and yielded theoretical shelf-life estimates of 31.9 h (25 °C), 104.7 h (15 °C), and 261.4 h (4 °C). These values closely matched observed shelf life, with relative errors within 3%. The Arrhenius analysis of the Logistic rate constants produced an apparent activation energy of 65.66 kJ·mol^−1^, indicating strong temperature sensitivity of sensory deterioration. Although leave-one-temperature-out validation revealed substantial uncertainty due to the limited number of calibration temperatures, the Logistic–Arrhenius framework provides a practical and interpretable basis for shelf-life estimation of packaged enoki mushrooms under constant-temperature storage, with clear potential for further refinement using more temperature points and dynamic cold-chain profiles.

## Figures and Tables

**Table 1:** Temperature-specific logistic model parameters and model-derived shelf-life estimates for *F. filiformis*.

Temperature (°C)	β_0_	k (h^−1^)	Theoretical Shelf Life, SL (h)	AIC
25	−11.081	0.3478	31.9	8.86
15	−11.913	0.1137	104.7	12.82
4	−12.066	0.0462	261.4	13.33

Note: $SL=-\beta_{0}/k$, corresponding to the time when p(OQ≥3)=0.5.

**Table 2:** In-sample agreement between observed shelf life and Logistic–Arrhenius-predicted shelf life of *F. filiformis* at the three calibration temperatures.

Temperature (°C)	Observed SL (h)	Predicted SL (h)	Relative Error (%)	Absolute Error (h)
25	31.1	31.9	+2.49	0.77
15	106.9	105.0	−1.91	2.04
4	264.0	261.4	−1.15	3.03

Note: Observed SL was obtained by linear interpolation from the measured $p\left(OQ\geq 3\right)$ data. Predicted SL was calculated from the fitted Logistic–Arrhenius model parameters.

## Data Availability

The original contributions presented in this study are included in the article/Supplementary Material. Further inquiries can be directed to the corresponding authors.

## Supplementary Materials

The following supporting information can be downloaded at: https://www.mdpi.com/article/10.3390/foods15132276/s1, Figure S1: Reference images for sensory scoring of enoki mushroom quality attributes (0–4 scale). Representative images used as visual references for sensory evaluation scoring (0–4 scale) during storage of *Flammulina filiformis*. Scores indicate increasing severity of quality deterioration, where 0 represents no visible defect (fresh appearance) and 4 represents severe deterioration. These reference images were used to assist panelists in assigning consistent scores to sensory attributes during evaluation. Table S1: Overview of enoki mushroom measurements and sampling scheme; Table S2: Sensory scoring criteria for enoki mushroom quality (0–4 scale); Supplementary Methods: Interpolation for spider-plot visualization.
