# Supplementary material for: A Sensory-Centered Logistic–Arrhenius Framework for Shelf-Life Prediction of Flammulina filiformis Under Different Storage Temperatures
Source: Foods. 2026 Jun 25;15(13):2276. doi: 10.3390/foods15132276 (PMC13361305; doi:10.3390/foods15132276)
Supplement: Supplementary file 1 [file foods-15-02276-s001.zip › foods-4383377-supplementary.pdf]

Table S1. Overview of enoki mushroom measurements and sampling scheme

Metric	Unit	Repeated measures	Sample unit
Cap color yellowing/dulling (A)	Rank, 0–4	Yes	Individual panelist score
Cap integrity loss (B)	Rank, 0–4	Yes	Individual panelist score
Surface sliminess/exudate (C)	Rank, 0–4	Yes	Individual panelist score
Off-odor (O)	Rank, 0–4	Yes	Individual panelist score
OVQ_{raw}=max(A,B,C)	Rank, 0–4	No (derived)	Derived per panelist record
OQ_{raw}=max(A,B,C,O)	Rank, 0–4	No (derived)	Derived per panelist record
p(OQ⩾3)	Proportion, 0–1	No (derived)	Temperature × time aggregate
Whiteness	%	Yes	New sample
Weight loss	%	Yes	New sample
PPO activity	U g^{-1}	Yes	New sample (destructive sampling)
MDA content	nmol g^{-1}	Yes	New sample (destructive sampling)
Soluble protein content	g kg^{-1}	Yes	New sample (destructive sampling)

Notes: Storage temperatures: 4, 15, and 25 °C. Sampling intervals: every 48 h (4 °C), every 24 h (15 °C), and every 12 h (25 °C). Objective indices were measured on newly sampled materials at each time point (no repeated measurements on the same sample). Shelf-life endpoint: $p(OQ \geq 3) \geq 0.5$).

Table S2. Sensory scoring criteria for enoki mushroom quality (0–4 scale)

Indicator	Score 0	Score 1	Score 2	Score 3	Score 4
O: Off-odor	Fresh, no off-odor: no abnormal smell, or only normal mushroom aroma	Very slight: detectable only when sniffing closely; not obvious away from the sample	Slight: detectable under normal sniffing, but still acceptable to most panelists	Moderate off-odor: clearly detectable, with a clear decline in acceptability (borderline; many panelists become unwilling)	Severe off-odor (unacceptable): strong, abnormal odor, clearly rejected
A: Cap color yellowing/dulling	Pure white, uniform	Slight yellowing (local pale yellow or slightly overall yellow tint)	Visible yellow patches or overall creamy-yellow tone; reduced uniformity	Obvious yellowing/dulling (clear beige/yellow tone)	Severe yellow-brown discoloration/dulling (clearly affects acceptability)
B: Cap morphology integrity	Caps are round, full, and uniform in size	Slight flattening or minor size non-uniformity	Partial flattening/irregular cap edges, with slight damage	Most caps flattened or opened; obvious damage/collapse	Severe deformation or damage; poor integrity
C: Surface sliminess/exudate	Surface is dry and non-glossy	Slight dampness/gloss; no clear adhesion or exudate	Visible damp gloss; slight adhesion or spot-like exudate	Obvious wet stickiness; sheet-like adhesion or visible exudate	Severe wet stickiness/exudate (clear water release)

Supplementary Methods: Interpolation for spider-plot visualization

For spider-plot visualization, continuous daily values between measured time points were estimated by linear interpolation to facilitate temporal comparison across storage temperatures with different sampling intervals. Interpolation was used only for visualization and did not replace observed values in statistical analyses or model fitting. The interpolated value $y$at day $x$was calculated from the two nearest measured points (x_1, y_1) and (x_2, y_2) as follows:

$$y = y_1 + \frac{(x - x_1)(y_2 - y_1)}{x_2 - x_1}$$

To enable visualizations of data along the same scale, min-max scaling was used.

$$X_i = \frac{X_i - X_{min}}{X_{max} - X_{min}}$$

Where *Scaled* $X_i$is the scaled value, $X_i$is the original value, $X_{min}$is the minimum value and $X_{max}$is the maximum value within a metric across all samples in the experiment.

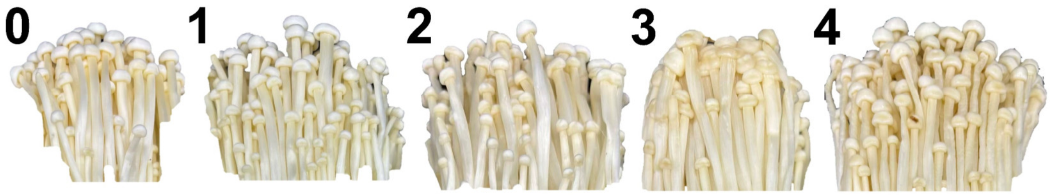

Figure S1. Reference images for sensory scoring of enoki mushroom quality attributes (0–4 scale). Representative images used as visual references for sensory evaluation scoring (0–4 scale) during storage of *Flammulina filiformis*. Scores indicate increasing severity of quality deterioration, where 0 represents no visible defect (fresh appearance), and 4 represents severe deterioration. These reference images were used to assist panelists in assigning consistent scores to sensory attributes during evaluation.
